# Validation of the Olfato-UP olfactory test for the Brazilian population

**DOI:** 10.1016/j.bjorl.2026.101808

**Published:** 2026-04-11

**Authors:** Denis Massatsugu Ueda, João Vitor Bazzo Chiesa, Leonardo Honório de Souza, Natália Medeiros Dias Lopes, Ellen Cristine Duarte Garcia, Marco Aurélio Fornazieri

**Affiliations:** a Universidade Estadual de Londrina (UEL), Department of Clinical Surgery, Londrina, PR, Brazil; b Pontifícia Universidade Católica do Paraná, Department of Medicine, Londrina, PR, Brazil

**Keywords:** Olfaction disorders, Smell, Anosmia, Reproducibility of results, Sensitivity and specificity

## Abstract

**Objective:**

To validate the Olfato-Up test in adults and elderly individuals from the Brazilian population.

**Methods:**

The study was conducted in three stages. The first phase evaluated the population’s familiarity with the chosen scents. The second phase assessed the performance of the Olfato-Up test, including sensitivity, specificity, accuracy, and cut-off determination. The third phase evaluated the test’s reproducibility and the time required for its application.

**Results:**

All fragrances met the minimum accuracy requirements for the normosmic population. The cut-off score for anosmia and severe hyposmia was 7, with a sensitivity of 88.2%, specificity of 86.6%, accuracy of 86.7%, and an Area Under the Curve (AUC) of 90.7% for anosmia. For severe hyposmia, the sensitivity was 70.2%, specificity was 96%, accuracy was 89.8%, and the AUC was 90.1%. The Intraclass Correlation Coefficient (ICC) was 0.89. The Pearson correlation test revealed a correlation coefficient of*r* = 0.811 (p = 0.001) and Bland-Altman test indicated a mean difference of −0.18 (95% CI −1.68 to 1.32). The average application time was 3.8 min (SD = 0.79).

**Conclusion:**

Olfato-Up test demonstrated excellent reproducibility, a short application time, and high sensitivity, specificity, and accuracy for detecting anosmia and severe hyposmia.

**Level of evidence:**

2.

## Highlights

• Olfato-Up is a new Brazilian olfactory test designed for cost reduction and reusability.
• The test demonstrated high reproducibility, sensitivity, specificity, and accuracy.
• Validation was conducted in the Brazilian adult and elderly population.

## Introduction

The ability to smell is primarily associated with hedonic situations such as enjoying a drink, food, and perfumes. However, it is also an essential sense for safety against environmental risks, being able to alert to exposure to toxic agents and spoiled food.^1^ Thus, olfactory disorder can lead to negative consequences varying degrees in the patient's quality of life.^2^ Among olfactory alterations, there is the possibility of distorted smells, odors that are not present in the environment, and the reduction of smell, whether partially or completely, parosmia and anosmia, respectively.

Regarding olfactory threshold, its evaluation gained prominence with the advent of COVID-19, as it was one of the initial symptoms of SARS-CoV-2 infection at the onset of the pandemic. Hyposmia is also related to pathologies in various medical fields beyond otolaryngology, such as dermatology, psychiatry, infectious diseases, and neurology.^3^, ^4^, ^5^, ^6^, ^7^ In this regard, it is important to highlight the need for evaluation with validated psychophysical tests for correct diagnosis, a patient's report of smell loss does not necessarily indicate reduced olfactory capacity, and conversely, hyposmia may occur in individuals without such complaints.^8^, ^9^, ^10^ Therefore, the use of validated tests is necessary for the correct management and investigation of patients with olfactory complaints.

Currently, several olfactory tests have been developed, validated, and are globally used. In Brazil, validated tests include Sniffin’ Sticks (Burghart Messtechnik GmbH, Wedel, Alemanha); Connecticut Chemosensory Clinical Research Center Test – CCCRC (Connecticut Chemosensory Clinical Research Center, Universidade de Connecticut, EUA); University of Pennsylvania Smell Identification Test (UPSIT®, Sensonics Inc., Haddon Heights, EUA) and Multiscent-20 (Noar, Brasil).^11^, ^12^, ^13^, ^14^, ^15^ However, for clinical practice in Brazil, the use of these tests faces financial or logistical barriers due to their high costs or long application times. For example, the average application time for UPSIT® is 10–15 minutes, and for CCCRC, it is 15–20 minutes.^16^, ^17^, ^18^

In order to find solutions for the time spent on tests, the Brief Smell Identification Test, previously known as the Cross-Cultural Smell Identification Test, was developed, with an estimated time of less than 5-minutes to complete the entire evaluation.^17^ Following the same objective, alternatives olfactory evaluation instruments have been developed and validated in other countries but not in Brazil. Olfactory tests developed in foreign countries require validation and, if necessary, adaptation to the country where they will be used since cultural aspects influence the identification of prevalent or non-prevalent odorants in the region.^17^^,^^19^^,^^20^ Regarding prices, the Connecticut Chemosensory Clinical Research Center (CCCRC) was validated for Brazil in 2022, and its commercialization is already taking place at present, with more accessible prices. Although compounding pharmacies and small companies in Brazil are increasingly able to produce butanol dilutions and prepare vials with odorants, availability remains heterogeneous across regions, which may still represent a logistical limitation.^16^^,^^18^ Multiscent-20 was recently validated in Brazil, being faster, containing 20 odors, but despite being national, its cost is also high due to the technology involved.^15^

Thus, currently, there is no test that solves the logistical, financial, and validation difficulties in the national territory. In view of these factors, Olfato-UP was created, the first olfactory test developed in Brazil that seeks to overcome the challenges presented. However, this test lacks validation in the national territory. Therefore, in this study, we validate Olfato-UP, evaluating minimum acceptable accuracy levels for each essence chosen by the developer, normalization table, application time analysis, and replicability.

## Methods

This is a cross-sectional study approved by the Ethics Committee of the Pontifícia Universidade Católica do Paraná (PUC/PR) under opinion number 58183422.0.0000.0020. Prior to their participation, individuals received and signed the informed consent form containing study information, risks, and benefits.

### Stages of the study

#### Phase 1 ‒ Confirmation of recognition index for the odorants used

In order to assess the familiarity of the population under analysis with the scents used in Olfato-UP, an evaluation was conducted on 175 volunteers aged between 18 and 87 years old, without olfactory complaints. The participants were divided into three age groups: 18–40-years old, 41–60 years old, and 61-years-old or older, and by sex. The Olfato-UP test was administered, and the percentage of correct responses for each item was evaluated in order to ensure an advisable index of 75% or more correct answers for each scent used.^12^ No adverse effects were reported.

#### Phase 2 ‒ Development of normative data

This phase involved 196 individuals (116 women and 80 men), with and without diagnosed olfactory loss assessed with the UPSIT®, to identify the cutoff point, evaluate sensitivity, specificity, accuracy, positive predictive value, and negative predictive value.

#### Phase 3 ‒ Test reliability

A retest was conducted on 32 volunteers from the first phase after a minimum interval of two weeks to assess the agreement between the results of both the test and the examination time.

### Patient selection

The 175 participants from phase 1 were volunteers without sinonasal problems, over 18-years-old, and with completed high school education. For phase 2, both patients with and without nasosinusal complaints were included as long as they met the criteria of adulthood and completed high school education. Individuals from phase 1 were randomly selected for retesting in phase 3.

Exclusion criteria for phase 1 included neurological disorders such as neurodegenerative and traumatic pathologies, epilepsy, stroke, neoplasms, chronic or acute rhinosinusitis (within the last 2-weeks), granulomatous nasal diseases, smoking, pregnancy, use of inhaled drugs and recent nasal surgery (less than 3-months). In phase 3, only participants who met any exclusion criteria between evaluations were excluded. There were no exclusion criteria for phase 2, as patients with or without complaints of hyposmia were included in the analysis.

### Procedures and tests

All participants were evaluated in a ventilated environment without any odor that could influence, such as air fresheners or cleaning products with scents. The order of administration of the Olfato-UP test and UPSIT® was randomized.

### Olfato-UP test

**Fig. 1:**
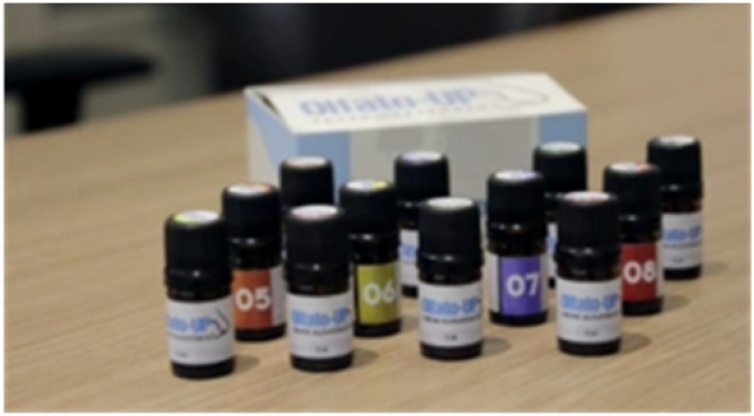
Olfato-UP test and its 12 bottles. Each bottle contains a specific odor.

**Fig. 2:**
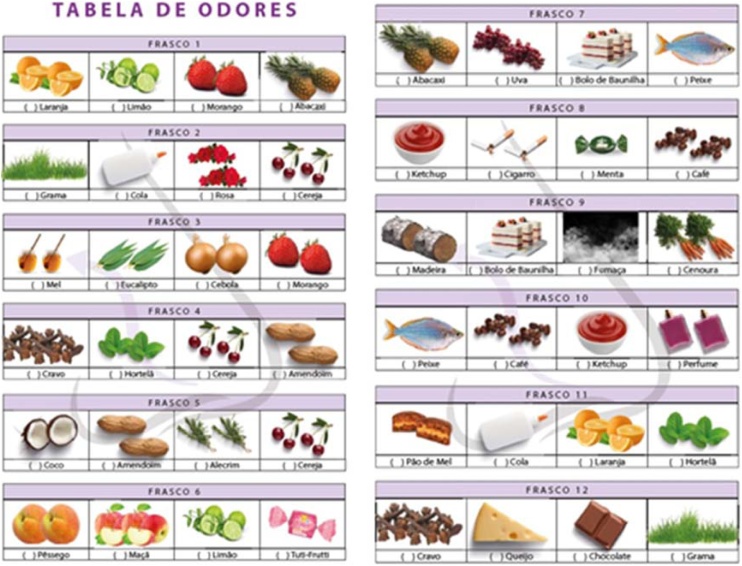
Olfato-UP test response card. Each alternative includes a word and its corresponding illustration.

Olfato-UP is a reusable, administered by an examiner or self-administered test. It is composed by 12 numbered vials containing one essence each for identification (Fig. 1). The odors are stored in 5 mL amber glass vials, each containing a piece of cotton onto which 0.5 mL of the essence is applied (the odors used are described in Supplementary Table 1). The patient is exposed to the odor by bringing the vial to within 1 centimeter of the nose. Then, it is necessary to mark on the response card the option that best identifies with the essence (Fig. 2). About the response card, the alternatives consist of words and corresponding images. The exam odorants are lemon, rose, eucalyptus, clove, rosemary, tutti-frutti, vanilla cake, coffee, wood, perfume, mint, and chocolate. Response card includes both words and corresponding images.

**Table 1:** Percentage of correct answers according to age group and gender in phase 1.

Odor	18‒40 Males (n = 35)	18‒40 Females (n = 35)	41‒60 Males (n = 26)	41‒60 Females (n = 30)	> 60 Males (n = 26)	> 60 Females (n = 23)
1 Lemon	77%	88.5%	76%	80%	76%	78%
2 Rose	77%	88.5%	76%	77%	76%	78%
3 Eucalyptus	100%	100%	96%	100%	100%	91%
4 Clove	100%	100%	100%	100%	100%	95%
5 Rosemary	100%	77%	84%	97%	88%	86%
6 Tuti-Frutti	97%	94%	100%	97%	96%	91%
7 Vanilla’s Cake	100%	94%	92%	97%	92%	82%
8 Coffe	88%	97%	76%	93%	76%	78%
9 Wood	77%	82%	76%	77%	76%	78%
10 Perfume	82%	80%	80%	80%	76%	86%
11 Min.	100%	97%	96%	93%	96%	91%
12 Chocolate	88%	100%	88%	86%	76%	78%

### University of Pennsylvania smell identification test

The University of Pennsylvania Smell Identification Test (UPSIT®) consists of four cards containing 10 odors each. The odorants are present in microcapsules, one on each sheet, and the aromas are released after scraping the area indicated by a brown stripe in the lower right corner. The test can be performed by the patient themselves or by an examiner. Based on the score achieved, it is possible to objectively classify the patient into normosmia, mild, moderate, and severe microsmia, and anosmia. Considered today one of the gold standard tests and already validated for the Brazilian population.^21^

### Statistical analysis

Descriptive analyses for quantitative data that showed normal distribution were performed, presenting the means accompanied by their respective Standard Deviations (±SD). Quantitative data without normal distribution were expressed using medians and Interquartile Range (IQR) (25%–75%). The assumptions of normal distribution in each group and homogeneity of variances between groups were assessed respectively using the Shapiro-Wilk test and Levene's test. Qualitative variables were presented as frequencies and percentages. To compare the Olfato Up’s score between groups of patients classified with normosmia, hyposmia (mild, moderate, and severe), and anosmia by UPSIT®, the Kruskal-Wallis test followed by Dunn's Test was used. The Receiver Operating Characteristic (ROC) curve was used to perform graphical analysis of sensitivity and specificity and determine the cut-off point with the highest sensitivity and specificity of the UPSIT® smell score.

The agreement between the test and retest scores of the olfactory score was assessed using the Intraclass Correlation Coefficient (ICC), and the guidelines for interpreting ICC were considered: <0.40 indicates poor agreement, 0.41‒0.60 indicates fair agreement, 0.61‒0.75 indicates good agreement, >0.75 indicates excellent agreement.^22^ For further assessment of Olfato-UP reproducibility in conjunction with the CCI, Pearson correlation coefficient and Bland-Altman test were performed. The analysis of test-retest time and percentage of correct answers between genders was conducted using the *t*-test. A Type I error probability (α) of 0.05 was considered in all inferential analyses. Descriptive and inferential statistical analyses were performed using SPSS version 22 (Armonk-NY, United States) and GraphPad version 8 (Boston-MA, United States).

## Results

### Phase 1

All scents met the minimum accuracy requirement of 75% (Table 1). Lemon and rose were the items with the lowest accuracy rates, contrasting with mint, which yielded results all above 90%. There was no difference in the percentage of correct responses between male and female genders in the age groups 18–40 years (p = 0.93), 41–60 years (p = 0.28), and over 60-years (p = 0.78).

### Phase 2

**Fig. 3:**
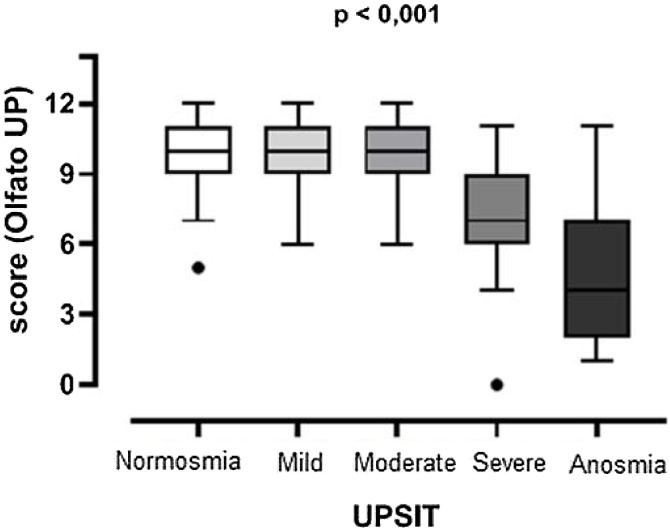
Box plot of Olfato-UP test scores in patients with normosmia, mild hyposmia, moderate hyposmia, severe hyposmia, and anosmia. UPSIT®, University Pennsylvania Smell Identification Test.

In comparing the Olfato-Up score among the normosmia, mild hyposmia, moderate hyposmia, severe hyposmia, and anosmia groups diagnosed by the UPSIT®, there was no difference in the score of Olfato-Up between quartiles 1, 2, and 3 for the categories of normosmia, mild, and moderate hyposmia. Dunn's test revealed a statistically significant difference between the severe hyposmia, and anosmia groups compared to the other categories (p < 0.001) (Table 2 and Fig. 3).

**Table 2:** Descriptive analysis of the median and quartiles of the normosmia, mild hyposmia, moderate hyposmia, severe hyposmia and anosmia groups.

UPSIT® classification	Median	IQR (25 %–75 %)
Normosmia	10	9.0–11.0
Mild hyposmia	10	9.0–11.0
Moderate hyposmia	10	9.0–11.0
Severe hyposmia	7	6.0–9.0
Anosmia	4	2.0–7.0

UPSIT®, University of Pennsylvania Smell Identification Test; IQR, Interquartile Range.

**Fig. 4:**
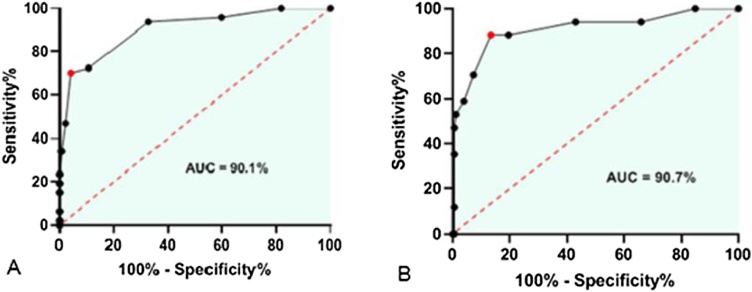
Receiver Operating Characteristic (ROC) curve illustrating the analysis of severe hyposmia (A) and anosmia (B).

The sensitivity and specificity analysis of Olfato-UP for patients with mild and moderate hyposmia showed poor performance in sensitivity, specificity, and accuracy. However, for severe hyposmia and anosmia, all the parameters studied yielded excellent results with an AUC of 90.1% for severe hyposmia and 90.7% for anosmia (Fig. 4 and Table 3). A cut-off of 7 showed the best balance of specificity, sensitivity, and accuracy for anosmia and severe hyposmia (Table 3).

**Table 3:** Sensitivity, specificity, Positive Predictive Value (PPV), Negative Predictive Value (NPV) and accuracy of identification of severe hyposmia and anosmia by the Olfato-UP test with 7 as cut-off.

	Anosmia		Severe hyposmia	
	%	95% CI	%	95% CI
Sensitivity	88.2	65.6 – 97.9	70.2	57.1 ‒ 83.3
Specificity	86.6	80.8 – 90.8	96.0	92.8 – 99.1
PPV	38.4	24.9 – 54.1	84.6	73.3 – 95.9
NPV	98.7	95.5 – 99.7	91.1	86.6 ‒ 95.5
Accuracy	86.7	82.0 – 91.5	89.8	85.6 ‒ 94.0

CI, Confidence Interval.

### Phase 3

Regarding the duration of the test application, the mean time for patients was 3.87-minutes (SD = 0.79) on the test and 3.62-minutes (SD = 0.60) on the retest, with an average time difference between test and retest of 0.21-minutes (95% CI 0.019 to 0.418; p = 0.03), showing a slight improvement in performance when evaluating for the second time.

**Fig. 5:**
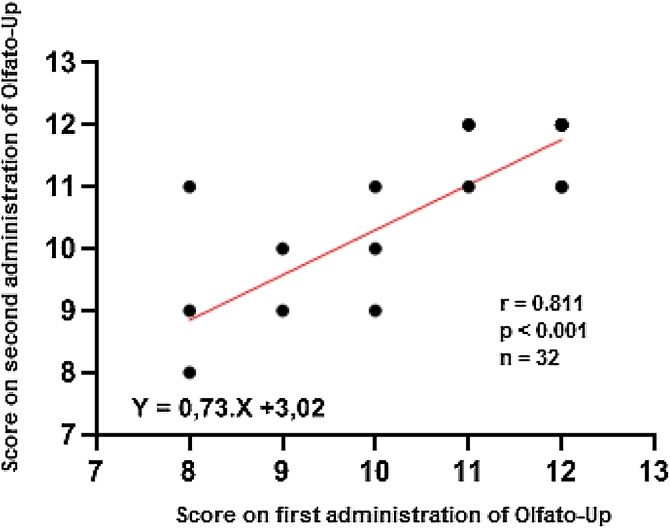
Test-retest correlation of Olfato-UP scores. The scatter plot shows a strong correlation (*r* = 0.811, p < 0.001, n = 32) between the first and second test administrations, with a linear regression line indicating agreement.

**Fig. 6:**
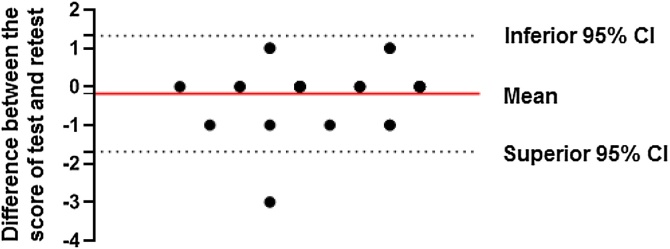
Agreement between test and retest scores of the Olfato-UP test.

The test and retest analysis had an intraclass correlation coefficient of 0.89 (95% CI 0.78‒0.94; p < 0.001), indicating excellent agreement.^21^ This result is consistent with the Pearson correlation test (*r* = 0.811; p < 0.001) (Fig. 5) and the Bland-Altman test (mean difference −0.18; 95% CI −1.68 to 1.32) (Fig. 6).

## Discussion

In the present study, it was possible to validate the Olfato-UP test (in adults and elderly individuals from the Brazilian population. The test demonstrated excellent sensitivity, specificity, and accuracy in detecting patients with anosmia. Additionally, the average time spent of 3.8-minutes (SD = 0.79) contributes to increased inclusion of olfactory assessments in medical consultations.

The selection of odors for the Olfato-UP test was based on Dimmick's study and the primary odors from the sterochemical theory^23^^,^^24^ aiming to encompass the categories proposed by these respective researches. Additionally, a criterion for selection was the presence of these essences in the daily lives of the Brazilian population, in order to use familiar odors for the patients.Regarding the substances used in olfactory tests and their means of delivery, there are various possibilities and methods, such as essences contained in plastic microcapsules like in the UPSIT®, or the product itself such as foods or cleaning articles.^13^^,^^14^ For Olfato-UP, it was chosen to use volatile oil in bottles due to its longer-lasting olfactory potential compared to the product or food itself, ease of transport, and low technological requirement for containing the essence, consequently resulting in lower overall costs. For use in Brazil, the Olfato-UP is considerably less expensive compared to the UPSIT®. While the UPSIT is an imported test, sold in U.S. dollars at a high price and subject to additional import fees and longer delivery times, the Olfato-UP is produced locally, available in Portuguese, and offered at a more affordable cost without import charges. This difference makes Olfato-UP a cost-effective alternative for clinical studies and large-scale applications, being particularly useful for patient screening within the Brazilian context and public health services.

Due to the higher prevalence of hyposmia in elderly patients and the difference in performance in some olfactory tests between men and women, we divided participants into groups by age and sex in evaluating the minimum percentage of correct responses to the used fragrances.^25^^,^^26^ All fragrances achieved the required accuracy rates. This is an important find to confirm the population’s familiarity with the smell used. Eucalyptus and clove showed high accuracy rates, while lemon, rose, and wood resulted in rates close to 75%. We believe that the variation in performance for these odors is due to the alternatives used, where distractor items are quite distinct for clove and eucalyptus, and some alternatives are similar for lemon, rose, and wood. Based on the pattern observed in our data, it is plausible that the identification rates for lemon, rose, and wood would increase if their distractors were composed of odor options that were more perceptually distinct from the target scent. Odors with highly dissimilar alternatives, such as clove and eucalyptus, showed the highest accuracy rates, supporting this interpretation. However, it is also important to consider that using distractors that are overly distant from the target odor may artificially inflate performance and reduce the ecological validity of the test. Future iterations of the Olfato-UP may benefit from a systematic re-evaluation of distractor sets to achieve an optimal balance between discrimination difficulty and representativeness of real-world olfactory demands.

Regarding the selection of distractor alternatives, these were not chosen based on the same theoretical frameworks used for defining the target odorants, such as Dimmick’s^24^ classification or the stereochemical theory of olfaction. Instead, distractors were selected empirically, prioritizing odors familiar to the Brazilian population, the availability of essences with stable olfactory characteristics, and the need to avoid options that were excessively obvious or unrelated to the target odor category. As a result, distractor selection was driven primarily by practical and cultural considerations rather than strict theoretical criteria. This approach may have contributed to variability in identification accuracy across certain items and highlights an opportunity for future refinement of the test structure.

Although volatile oils were selected for the Olfato-UP due to their inherently longer odorant stability compared to food-based or microencapsulated products, we acknowledge the importance of objectively documenting this characteristic. A formal chemical stability test was not performed as part of this study; however, a practical assessment was conducted during routine test administration. Under standard storage conditions (sealed bottles, room temperature, and protection from light), the odorants retained adequate perceptual intensity for approximately six months, based on weekly inspections by the research team. Therefore, we recommend replacing the vials every 180 days for clinical use. Future investigations will incorporate controlled stability analyses to establish standardized degradation profiles for each essence.

With a focus on accessibility, the Olfato-UP response card includes both words and corresponding images. Previous studies suggest that answer sheets containing only words in the alternatives do not yield different results from those containing words and drawings.^12^^,^^27^ However, due to the limited literature on the topic, it was decided to retain the written alternatives and their corresponding figures to encompass alternative pathways of understanding for non-literate patients.

For normative data, a cut-off of 7 was chosen for anosmia, with sensitivity at this point being 88.2% (95% CI 65.6–97.9), specificity 86.6% (95% CI 80.8–90.8), and accuracy 86.7% (95% CI 82.0–91.5). Therefore, a result of six or less would indicate anosmia for Olfato-Up test. Regarding the ROC curve for anosmia, the AUC is 90.7%, considered an excellent result.^28^ It is interesting to highlight the intraclass correlation coefficient of 0.89, the Pearson correlation coefficient (*r* = 0.811; p < 0.001) and Bland-Altman test mean −0.18; 95% CI −1.68 to 1.32), representing excellent reproducibility with all three tests.^22^^,^^29^^,^^30^

Despite Olfato-UP test performance being good for identifying patients with severe hyposmia and anosmia, there is difficulty in identifying those with mild to moderate hyposmia, as these groups did not show significant differences compared to normosmic volunteers. It is important for future studies to focus on improving the test to also differentiate patients with mild to moderate hyposmia between those without olfactory problems. Future studies will also be useful to validate the test for the child population.

Among the strengths of the research, the definition of the cut-off based on the ROC curve and without arbitrariness stands out, as well as the large sample encompassing young adults to elderly and the analysis of time to confirm the necessary test application time. The Olfato-UP can contribute to speeding up the diagnosis of patients with olfactory loss, reducing the need to extend the investigation in cases of anosmic patients. However, it is worth noting that there is room for improvement in the test to encompass other categories of olfactory reduction.

## Conclusion

The Olfato-UP is a cost-effective, quick, reusable, and self-administered test designed to simplify olfactory assessment in medical care. Now validated for the Brazilian population aged 18 and above, it offers a practical and quickly option for olfactory evaluation.

## ORCID ID

Denis Massatsugu Ueda: 0000-0002-6587-947X

João Vitor Bazzo Chiesa: 0009-0004-7287-4951

Leonardo Honório de Souza: 0009-0002-4545-4535

Natália Medeiros Dias Lopes: 0000-0001-6457-7552

Marco Aurélio Fornazieri: 0000-0001-5213-2337

## Funding

This research did not receive any specific grant from funding agencies in the public, commercial, or not-for-profit sectors.

## Data availability statement

The authors declare that all data are available in repository.

## Declaration of competing interest

Dr. Marco Aurélio Fornazieri is the creator of the Olfato-UP olfactory test and the founder and coordinator of the Medical Excellence Group (GEM). GEM holds the rights to the Olfato-UP test. All other members of GEM declare no conflicts of interest.
