# Supplementary material for: Validation of the Olfato-UP olfactory test for the Brazilian population
Source: Braz J Otorhinolaryngol. 2026 Apr 11;92(4):101808. doi: 10.1016/j.bjorl.2026.101808 (PMC13092605; doi:10.1016/j.bjorl.2026.101808)
Supplement: Supplementary file 1 [file mmc1.docx]

**BJORL-D-25-00057_Supplementary Material**

**Supplementary Table 1** Detailed specifications of odorants used in the Olfato-UP Test.

| **Odorant** | **Product Number** | **Manufacturer** |
| --- | --- | --- |
| 1 Lemon | XABQ3LM9N | Senes essências |
| 2 Rose | 98UK6QBDB | Senes essências |
| 3 Eucalyptus | 002457 | Quinarí |
| 4 Clove | 002339 | Quinarí |
| 5 Rosemary | 756296317 | Quinarí |
| 6 Tuti-Frutti | 7WVWV7Z82 | ATR essências |
| 7 Vanilla’s Cake | BAUNILHAHS | ATR essências |
| 8 Coffe | XM6ZUCB63 | ATR essências |
| 9 Wood | FSNATNGUH | ATR essências |
| 10 Perfume | HXS3PLJ7U | ATR essências |
| 11 Mint | MENTAHS | ATR essências |
| 12 Chocolate | LKVEDSHUL | ATR essências |

* Dilution: All scents were used undiluted.
